# Necrotizing Fasciitis Caused by Hypermucoviscous *Klebsiella pneumoniae* in a Filipino Female in North America

**DOI:** 10.5811/westjem.2014.11.23599

**Published:** 2014-12-05

**Authors:** Daniel Ng, Brad Frazee

**Affiliations:** Highland Hospital – Alameda Health System, Department of Emergency Medicine, Oakland, California

## Abstract

Necrotizing fasciitis caused by *Klebsiella pneumoniae* has been described in Southeast Asia, but has only recently begun to emerge in North America. The hypermucoviscous strain of *K. pneumoniae* is a particularly virulent strain known to cause devastatingly invasive infections, including necrotizing fasciitis. Here we present the first known case of necrotizing fasciitis caused by hypermucoviscous *K. pneumoniae* in North America.

## INTRODUCTION

*Klebsiella pneumoniae* is a member of the enterobacteriacea family of gram-negative rods that are found primarily in the human gastrointestinal tract. The most common forms of Klebsiella infection are hospital- acquired urinary tract infections, pneumonia, and bacteremia. Much of the recent literature surrounding *K. pneumoniae* has been in regards to its role as a carrier of extended spectrum beta-lactamase (ESBL), and more recently, as the predominant carbapenem resistant enterobacteriaceae (CRE) species. CRE is a new class of multi-drug resistant species that tend to infect elderly patients after prolonged hospital stays or in long-term care facilities.^1^ The substantial mortality associated with CRE infections is likely due the lack of effective treatments and underlying vulnerability of the patients, rather than virulence of the bacteria.^1^

This case report describes a strain of *K. pneumoniae*, referred to as hypermucoviscous, that is distinctly different from CRE. Hypermucoviscous *K. pneumoniae*, unlike CRE, is community acquired, highly virulent and essentially pansensitive.^2^ Hypermucoviscous *K. pneumoniae* causes invasive infections, including liver abscess, endopthalmitis, meningitis, empyema, and necrotizing fasciitis, that occur primarily in Southeast Asia. Here we report a case of monomicrobial necrotizing fasciitis caused by hypermucoviscous *K. pneumoniae* in a Filipino woman who presented to our public hospital in Oakland, California. While there have been limited reports of infection with this unusual *K. pneumoniae* strain outside of Asia, to our knowledge this is the first report of necrotizing fasciitis caused by confirmed hypermucoviscous *K. pneumoniae* in North America.

## CASE REPORT

A 71-year-old Filipino female with no known past medical history presented to an emergency department in Oakland, California, for neck swelling, fever, and difficulty breathing. She had been experiencing these symptoms for two weeks, with the neck swelling becoming progressively worse. On physical exam the patient appeared ill with a heart rate of 139, blood pressure of 87/36 and temperature 101.1, indicating septic shock. Physical exam revealed a large fluctuant mass over the left lateral neck. The center of this mass exhibited blackish discoloration and skin necrosis. Swelling and crepitus extended to the anterior and posterior neck, left shoulder and anterior chest wall.

**Figure 1:**
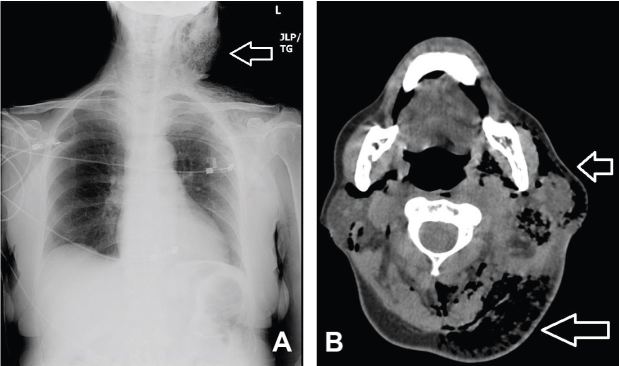
*A*, Chest radiograph and *B*, neck computed tomography image at level of C2, both demonstrating left-sided neck mass with extensive subcutaneous emphysema (open arrows).

Initial laboratory evaluation showed a white blood cell count of 22.9thou/mcL, Hemoglobin of 14.8g/dL, and platelets of 359thou/mcL. Notable chemistries were sodium of 125mmol/L, potassium 4.9mmol/L, chloride 110mmol/L, bicarbonate less than 5mmol/L, blood urea nitrogen 41mg/dL, creatinine 2.5mg/dL, glucose 917mg/dL, and lactic acid 3.5mmol/L. Urinalysis showed glucosuria and ketonuria. CT of chest and neck revealed extensive subcutaneous emphysema throughout the left lateral upper chest wall, left shoulder region, anterior mediastinum and throughout the superficial and deep spaces of the neck (Figure 1).

The patient was taken to the operating room for debridement and was discovered to have necrotic deep muscle tissue and fascia. Intraoperative biopsies confirmed the diagnosis of necrotizing fasciitis, with necrotic and purulent material found in the dermis, subcutaneous tissues, and fascia.

During the patient’s hospital stay, she required numerous vasopressors and steroids for refractory hypotension, hemodialysis for refractory acidosis and uremia, and was taken to the operating room for debridement a total of three times. The patient expired on her seventh hospital day due to overwhelming sepsis and acidosis.

Cultures of blood, urine, and surgical specimens all grew *K. pneumoniae*. These isolates were string-test positive, indicating that this was the hypermucoviscous strain. All cultures were resistant to ampicillin, but otherwise were pan susceptible.

## DISCUSSION

The distinctive clinical syndrome of invasive hypermucoviscous *K. pneumoniae*, consisting of liver abscesses, bacteremia, and metastatic infection, particularly of the central nervous system, is now well described. To a large extent, the syndrome has been geographically restricted to Southeast Asia. The association of this pathogen with a wider range of invasive infections, including soft tissue abscesses and necrotizing fasciitis has only been recognized more recently with the first description of necrotizing fasciitis appearing in 1996.^3^ While much of the existing literature consists of case reports,^4^–^8^ a recent large case series from Taiwan systematically evaluated *K. pneumoniae* necrotizing fasciitis.^9^ In this single hospital study, *K. pneumoniae* accounted for 17% of monomicrobial necrotizing fasciitis cases as compared to 22% due to *S. aureus* and 18% due to group A *Streptococcus*. Fifteen *K. pneumoniae* cases were compared to a similar number caused by group A Streptococcus – an organism more traditionally associated with necrotizing fasciitis. The investigators found that *K. pneumoniae* cases exhibited higher mortality and higher rates of bacteremia and that patients were more likely to be immunocompromised, with 80% having diabetes*.*

Outside of Asia, *K. pneumoniae* has just begun to emerge as a cause of necrotizing fasciitis. Three cases have been described in Europe.^10^–^12^ The first case of *K. pneumoniae* necrotizing fasciitis described in North America was in 2007, in which a Cambodian man with travel to Cambodia six months prior was diagnosed with necrotizing fasciitis and died in three days.^13^ Two subsequent reports described *K. pneumoniae* necrotizing fasciitis in patients who had no recent travel to Asia and were not of Asian descent.^14^,^15^ A recent North American case series reported on six liver transplant recipients, who developed *K. pneumoniae* necrotizing fasciitis, all of whom died.^16^ There is one report specifically of *K. pneumoniae* cervical necrotizing fasciitis, similar to our case, that required 12 surgical debridements with the patient surviving.^17^

**Figure 2:**
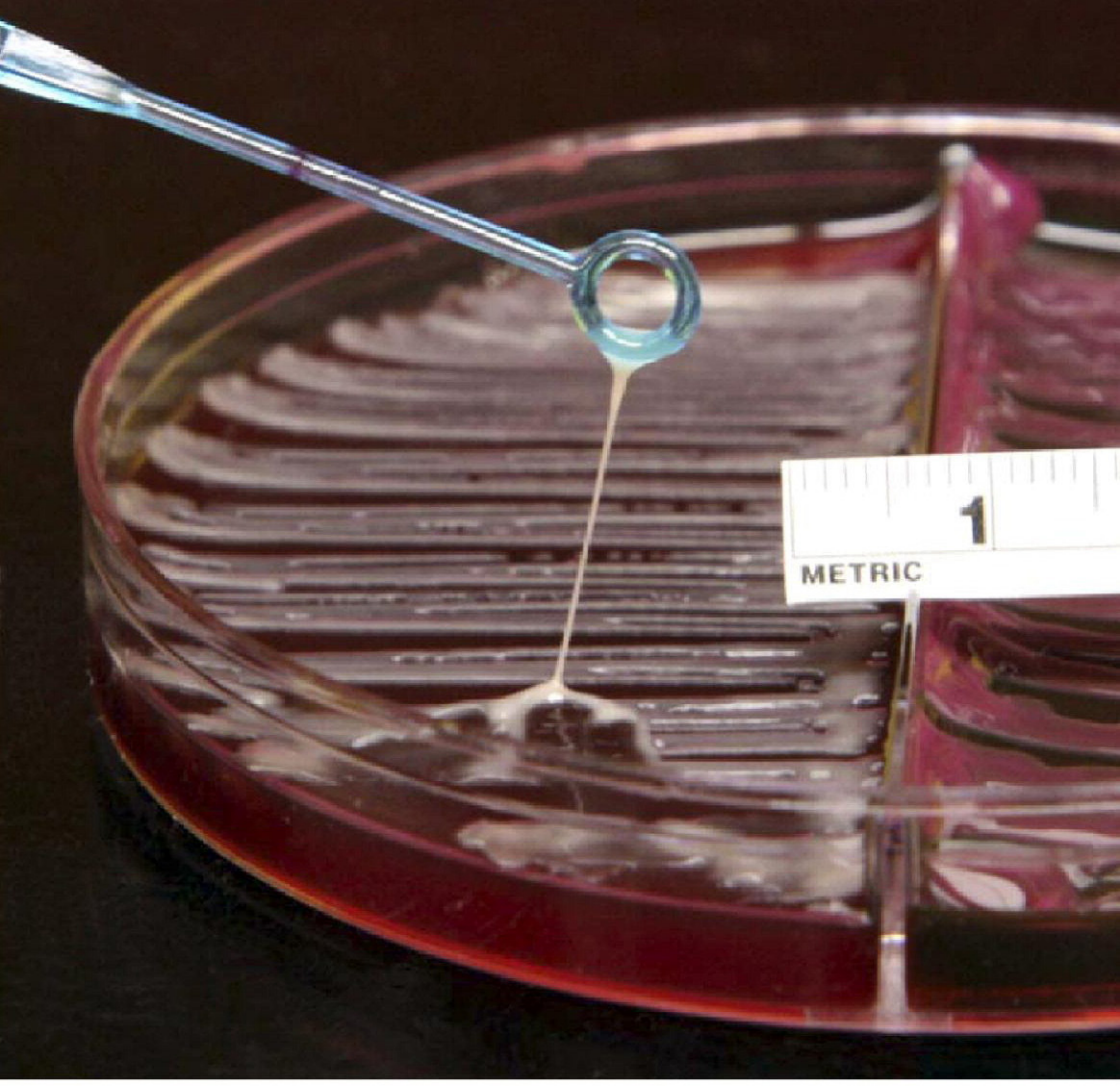
Example of a positive string test (>5mm string) indicating the hypermucoviscous phenotype.

Ours is the first case report of *K. pneumoniae* necrotizing fasciitis in the North America to confirm the hypermucoviscous phenotype. Hypermucoviscous strains are identified in the laboratory with a simple string test, in which a colony is lifted with a loop, producing a string longer than 5mm (Figure 2). The phenotype is associated with the rmpA and magA genes;^18^ although we did not perform genotyping in this case, we have previously shown the hypermucoviscous *K. pneumoniae* isolates from our hospital were rmpA positive.^19^ The hypermucoviscous phenotype is thought to confer virulence by a number of mechanisms, including its ability to resist phagocytosis, and both complement and neutrophil-mediated killing, and its ability to more efficiently acquire iron.^20^,^21^ These virulence factors lead to a destructive clinical syndrome, often with multiple infectious metastases.^20^–^22^ It is likely that most of the community-acquired invasive *K. pneumoniae* infections recently reported in North America, including the previously reported necrotizing fasciitis cases, were also due to the hypermucoviscous strain, but that string testing was simply not performed. Hypermucoviscous *K. pneumoniae* strains are invariably cephalosporin susceptible, so the finding of broad antibiotic susceptibility in a *K. pneumoniae* isolate from a community-acquired invasive infection represents indirect evidence that it is a hypermucoviscous strain. As expected, most of the studies of *K. pneumoniae* necrotizing fasciitis outside of Asia reported similar antibiotic susceptibility profiles that fit this pattern.^10^,^13^–^15^

We previously reported 13 cases of invasive infection caused by hypermucoviscous *K. pneumoniae*.^19^,^23^ In our series, multiple types of infectious were found, including neck abscesses, pyelonephritis, brain abscesses, pneumonia, liver abscesses, and cholecystitis. Including the current case, four patients with skin and soft tissue infections due to hypermucoviscous *K. pneumoniae* have been seen since 2007 at our urban public hospital in Northern California.

Interestingly, hypermucoviscous *K. pneumoniae* still remains largely confined to Asia and cases in North America have occurred disproportionately in patients of Asian descent. This has raised speculation as to a genetic susceptibility to colonization and/or infection.^21^ Data from stool samples from healthy Chinese and Korean adults residing throughout Asia have also suggested that a small percentage of Asians are colonized with hypermucoviscous *K. pneumoniae*.^21^ Alternatively, patients may simply acquire hypermucoviscous *K. pneumoniae* from living in or traveling to endemic areas. Regardless, it seems that being of Asian descent and recent travel to Asia are the most important risk factors, along with diabetes mellitus, for developing a hypermucoviscous *K. pneumoniae* infection.

Our West Coast urban safety net hospital serves a large Southeast Asian population, including recent immigrants, which likely accounts for the large number of cases we have seen. Yet it is also likely that hypermucoviscous *K. pneumoniae* has made an unrecognized emergence elsewhere in northern California and perhaps elsewhere in North America. While we predicted in 2009 that this pathogen was likely to emerge dramatically in the U.S., subsequent reports have been limited. Ultimately, routinely testing for and identifying hypermucoviscosity in *K. pneumoniae* isolates has limited clinical importance in changing early management, especially for necrotizing soft tissue infections, as aggressive sepsis care, early empiric broad spectrum antibiotics, and prompt source control remain priorities in the treatment of necrotizing soft tissue infections, regardless of etiology. On the other hand, microbiologic surveillance for emerging pathogens is a potentially important role of emergency departments, especially those located in communities with large immigrant populations.^24^,^25^ The appearance of a new and virulent pathogen here in North America will certainly have public health implications; however, it is difficult to predict what these might be, as confirmed cases are sparse in number and based on our suspicions, other cases are potentially not being recognized. We therefore advocate that string testing be performed routinely on all *K. pneumoniae* isolates. In addition to identifying this clinically distinctive syndrome and thereby prompting a search for metastatic infections, routine string testing of *K. pneumoniae* isolates might illuminate the connection with the Southeast Asian data and clarify whether this pathogen is emerging rapidly outside of Asia.
